# A word of caution

**DOI:** 10.1002/clc.23420

**Published:** 2020-07-11

**Authors:** Neelesh Gupta, Krunalkumar Patel, Rajeev Gupta

**Affiliations:** ^1^ Department of Internal Medicine Nazareth Hospital Philadelphia Pennsylvania USA; ^2^ Department of Internal Medicine St Mary Medical Center Langhorne Pennsylvania USA; ^3^ Department of Cardiology Mediclinic Al Jowhara Hospital Al Ain United Arab Emirates

To the Editor

We read with interest the review “Real‐world use of nonvitamin K antagonist oral anticoagulant (NOAC) in atrial fibrillation (AF) patients with liver disease: A meta‐analysis” by Qixin Dai et al.^1^ The meta‐analysis is informative as unlike clear‐cut guidelines for the use of anticoagulant drugs in patients with renal impairment the guidelines for patients with preexisting liver diseases are conspicuously missing. The study shows NOACs are not only safer but more efficacious than warfarin in patients with liver disorders with nonvalvular AF. A clinically important message as use of warfarin despite carrying more bleeding liabilities is not efficacious in patients with end‐stage renal impairment.^2^

The authors must cogently emphasize that bias is inevitable despite careful selection of the included studies; as they are retrospective, therefore despite adjusted analysis many unmeasured and residual variables remain unaccounted. The current regulatory recommendations are rather clear for patients with liver disease with Child‐Pugh A (warfarin or NOACs are equally safe and effective) and with score Child‐Pugh C (only warfarin is indicated); however, for patients with liver disease with Child‐Pugh B score, a sufficiently powered randomized control trial is the need‐of‐the‐hour to answer what is the best alternative warfarin or one of the NOACs (preferably either dabigatran or apixaban, largely because of more favorable pharmacodynamics and pharmacokinetics in patients with liver diseases).^3^
